# Combination of Anti-EGFR and Anti-VEGF Drugs for the Treatment of Previously Treated Metastatic Colorectal Cancer: A Case Report and Literature Review

**DOI:** 10.3389/fonc.2021.684309

**Published:** 2021-05-24

**Authors:** Yong Li, Xian Chen, Wenzhu Li, Yongsong Ye, Xiaohua Du, Shaodan Sun, Lirong Liu, Haibo Zhang

**Affiliations:** ^1^ Department of Oncology, Guangdong Provincial Hospital of Chinese Medicine, Guangzhou, China; ^2^ Department of Image, Guangdong Provincial Hospital of Chinese Medicine, Guangzhou, China; ^3^ Department of Pathology, Guangdong Provincial Hospital of Chinese Medicine, Guangzhou, China; ^4^ Deparment of Pharmacology of Traditional Chinese Medicine, Guangdong Provincial Hospital of Chinese Medicine, Guangzhou, China; ^5^ State Key Laboratory of Dampness Syndrome of Chinese Medicine, Guangdong Provincial Hospital of Chinese Medicine, Guangzhou, China

**Keywords:** resistance, cetuximab, fruquintinib, chemotherapy, metastatic colorectal cancer

## Abstract

The standard third-line treatment of metastatic colorectal cancer (mCRC) includes the small-molecule anti-vascular drugs (Regofenib and Fruquintinib) and the chemotherapy drug trifluridine and tipiracil hydrochloride (TAS-102). There is no standard treatment for mCRC if the third-line treatment failed. Therefore, it is a pressing need to develop new therapeutic approaches to improve the survival of patients who developed drug resistance to the third-line treatment. In this study, we report a case of mCRC with RAS/BRAF wild-type, who was successfully treated using cetuximab in combination with fruquintinib after resistance to chemotherapy, bevacizumab, cetuximab and regorafenib. This patient responded to this combination regimen. Then, we discuss the mechanisms of action of this combination. Furthermore, we introduce the clinical trials on the combination regimens of anti-EGFR with anti-vascular monoclonal antibodies. Finally, we discuss the clinical explorations of using combination of anti-EGFR with small-molecule anti-VEGF drugs and their potential benefits. The clinical effects of small-molecule anti-vascular drugs in combination with anti-EGFR in the treatment of CRC warrant further explored.

## Introduction

Therapeutic regimens for metastatic colorectal cancer (mCRC) include chemotherapy and targeted therapy. The chemotherapeutic drugs generally include fluorouracil, oxaliplatin, and irinotecan, while targeted drugs include the antiangiogenic drugs (bevacizumab, ziv-aflibercept, and ramucirumab) and anti-EGFR drugs (cetuximab or panitumumab for RAS, BRAF wild-type patients). With the application of these drugs and the development of predictive factors, the median overall survival (OS) of patients with mCRC has exceeded 30 months (1). However, the prognosis of patients who failed the first- and second-line treatments is still poor. At present, the third-line standard treatment of mCRC include small-molecule anti-vascular drugs, such as regorafenib and fruquintinib, and chemotherapeutic drugs, such as trifluridine and tipiracil hydrochloride tablets (TAS-102). For patients with RAS/BRAF wild-type who have not used the anti-EGFR drug, cetuximab in combination with irinotecan can be selected (2–6). For patients with rare mutations, including HER2 amplification, BRAF mutation, and MSI-H, corresponding targeted drugs (7) can be applied.

Currently, there is no standard treatment available after CRC patients develop drug resistance to the third-line treatment. The treatments for those patients include different combination of drugs and are in the exploratory stage. Anti-VEGF drugs combined with immune checkpoint inhibitors in proficient mismatch repair(pMMR) patients (8) or TAS-102 combined with Anti-VEGF drugs (9) are commonly used. Rechallenge of chemotherapy, cetuximab and the combination of anti-EGFR and anti-VEGF are alternatives.

Hear we report a case of advanced CRC who is RAS/BRAF wild-type and developed drug resistance to the previous therapy. This patient was treated successfully by a small-molecule anti-vascular drug in combination with cetuximab. The aim of this study is to present the mechanisms of action and clinical applications of anti-EGFR combined with anti-VEGF drugs, and discuss the potential benefits of this combination.

## Case Presentation

**Figure 1:**
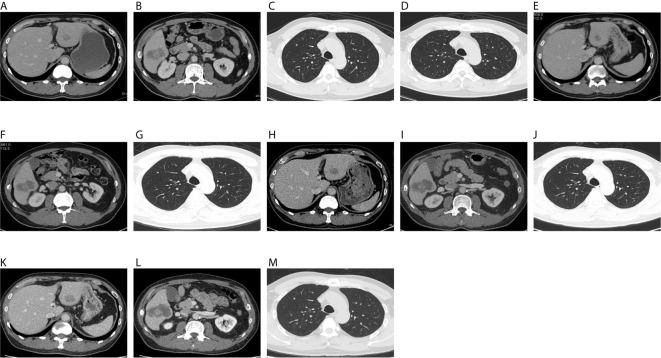
On December 18, 2019, CT showed hepatic S3 metastases with a long diameter of 1.8 cm **(A)**, hepatic S6 metastases with a long diameter of 4.4 cm **(B)**, right upper lung metastases with a long diameter of 0.3 cm **(C)**, and a metastatic tumor of left upper lung, with a long diameter of 0.25 cm **(D)**. On April 3, 2020, CT showed hepatic S3 metastases with a long diameter of 1.4 cm **(E)**, hepatic S6 metastases with a long diameter of 4.5 cm **(F)**, and right upper lung metastases with a long diameter of 0.3 cm **(G)**. The original left upper pulmonary nodule was not shown. On June 6, 2020, CT showed hepatic S3 metastases with a long diameter of 2.1 cm **(H)**, hepatic S6 metastases with a long diameter of 5.2 cm **(I)**, and right upper lung metastases with a long diameter of 0.3 cm **(J)**. The original left upper pulmonary nodule was not shown. On August 6, 2020, CT showed hepatic S3 metastases with a long diameter of 2.1 cm **(K)**, hepatic S6 metastases with a long diameter of 5.5 cm **(L)**, and right upper lung metastases with a long diameter of 0.3 cm **(M)**. The original left upper pulmonary nodule was not shown.

The patient was a 52-year-old male. In March 2017, the patient presented with hematochezia without obvious causes, and a mass of about 2.7 × 3.5 cm was found 35 cm from the anus through electronic colonoscopy. CT imaging showed a sigmoid colon neoplasm, invading to the serosa, local multiple lymph node metastases, liver, and lung multiple metastases. The biopsy confirmed the patient had moderately differentiated adenocarcinoma of the sigmoid colon. The blood level of carcinoembryonic antigen (CEA) and CA199 in this patient was normal. The tumor stage was diagnosed as T3N1M1b. The hepatobiliary surgeon assessed liver metastasis as unresectable. A genetic testing was performed and revealed wild type KRAS, NRAS, BRAF, and PIK3CA of the cancer. Immunohistochemical (IHC) staining showed positive for mutS homolog 2 (MSH2), mutS homolog 6 (MSH6), and mutl homolog l gene (MLH1), and weakly positive for PMS1 homolog 2 (PMS2). The DNA mismatch repair (MMR) was proficient mismatch repair (pMMR). IHC staining of HER2 was negative. According to the treatment guidelines, the first-line treatment should be chemotherapy combined with cetuximab. Because cetuximab was not included in the national medical insurance at that time, bevacizumab in combination with XELOX chemotherapy was applied as the first-line treatment from May to August 2017. The treatment efficacy was partial response (PR). The patient was followed with maintenance treatment using capecitabine. The efficacy was progressive disease (PD) in June 2018. Bevacizumab in combination with FOLFOX chemotherapy was introduced in August 2019, and the efficacy was PD in January 2019. Cetuximab in combination with irinotecan chemotherapy was started as the third-line treatment in January 2019. CT imaging indicated cancer progression in September 2019. Therefore, he was treated with regorafenib as the fourth-line therapy in October 2019 for two cycles. Reexamination indicated PD in December 2019. CEA level was 48.6 mmol/L and CA199 level was 39.3 mmol/L. Because TAS-102 is not approved in China, from January 30 to August 6, 2020, this patient received 11 cycles of cetuximab, 500 mg/m (2) biweekly, in combination with fruquintinib, 5 mg once daily for the first 3 weeks of each 4 weeks as the fifth-line therapy. During this period, reexamination by abdominal contrast-enhanced CT on April 3, 2020, indicated stable disease (SD) with tumor shrinkage. Reexamination by the chest and abdominal contrast-enhanced CT on June 6, 2020, indicated SD (**Figure 1**). CEA and CA199 levels were decreased to 20.33 mmol/L and 13.89 mmol/L, respectively. The last evaluation was conducted in August 2020, and the progression-free survival (PFS) was 8 months. During the treatment, the patient had elevated grade 1 aspartate aminotransferase and alanine aminotransferase, elevated grade 1 hypercalcemia, and grade 1 acne-like rash. There was no other obvious side effect.

## Current Status of the Third-Line Regimen

As an oral small-molecule multi-target kinase inhibitor, regorafenib inhibits multiple targets, including VEGFR1, VEGFR2, VEGFR3, TIE2, Kit, RET, PDGFR, and FGFR1 (2). The CORRECT trial and the CONCUR trial using the third-line therapy in CRC patients have shown that the median OS (mOS) of the regorafenib group is significantly better compared with the placebo group (6.4, 8.8 months vs. 5.0, 6.3 months, HR=0.77) (3, 4). Fruquintinib is a small-molecule antiangiogenic agent independently developed in China. The results of the FRESCO trial have indicated that the mOS of Chinese CRC patients treated with fruquintinib as the third-line therapy is significantly prolonged compared with the patients receiving placebo (mOS: 9.3 months vs. 6.6 months, HR=0.65) (5). TAS-102 is a new type of cytotoxic antineoplastic drug used in the third-line therapy. TAS-102 significantly prolonged the OS compared with the placebo group in advanced CRC patients (6, 10). Cetuximab is an anti-EGFR monoclonal antibody recommended to be used as a third-line therapy for anti-EGFR-therapy naïve, RAS wild-type advanced CRC patients (11). Cetuximab can be rechallenged in CRC patients resistant to cetuximab. It has been found that after the discontinuation of anti-EGFR monoclonal antibodies, *RAS* and *EGFR* relative mutant allele frequency decays exponentially (*r* ^2^ = 0.93 for *RAS*; *r* ^2^ = 0.94 for *EGFR*) with a cumulative half-life of 4.4 months. This finding supported the re-challenge of anti-EGFR monoclonal antibodies after the initial failure and guided the optimal timing of re-challenge initiation (12). A phase II single-arm study has reported a response rate of 21% for the rechallenged use of cetuximab. Patients with RAS wild-type ctDNA have significantly longer PFS than RAS-mutated patients (median PFS, 4.0 vs. 1.9 months) (13). The re-challenging regimen usually uses cetuximab plus irinotecan. However, only few studies have focused on the combined use of cetuximab and antiangiogenic agents.

## The Mechanisms of Combination of Anti-EGFR and Anti-VEGF Drugs

The VEGF and EGFR share the same downstream signal transduction components. Therefore, therapies targeting these two pathways may have additive or even synergistic therapeutic effects. Compared with the inhibition of a single pathway, the combination of VEGF antisense oligonucleotides and cetuximab to block VEGF and EGFR enhanced the antitumor activity and improve the survival rate in mice carrying CRC xenografts of human GEO colon cancer cells (14). Similarly, the combination treatment of cetuximab and antiVEGF-2 monoclonal antibodies improved the antitumor activity of mice with metastasis induced by the intraperitoneal injection of KM12L4 human colon cancer cells (15). Treatment with anti-EGFR monoclonal antibodies can inhibit VEGF production, while treatment with vandetanib (a tyrosine kinase inhibitor of VEGF receptors) can prevent epidermal growth factor-induced EGFR phosphorylation (16). Anti-VEGF and EGFR antibodies could not only reduce CRC cells proliferation and invasion *in vitro*, but also inhibit the tumor growth and angiogenesis *in vivo* through inhibiting the activation of AKT and ERK signaling pathways. This study partly explains the underlying mechanisms of the combinational use of anti-VEGF and anti-EGFR drugs (17). These preclinical studies with improved tumor inhibition may be attributed to elucidate the interaction between VEGF and EGFR signaling pathways.

Furthermore, anti-VEGF and anti-EGFR can also affect tumor microenvironment (TME). Cetuximab can stimulate antibody-dependent cell-mediated cytotoxicity (ADCC) and induce innate and adaptive immunity (18). Anti-VEGF can increase the infiltration of immune effector cells into tumors and convert the intrinsically immunosuppressive tumor microenvironment to an immune-supportive one (19). Regorafenib has the important effect of enhancing anti-tumor immunity *via* macrophage modulation and anti-immunosuppression by inhibiting CSF1R (20, 21). But their combinational effect on TME remains unknown. Therefore, the effect of this combination regimen on TME warrants further investigation.

## Clinical Trials on Combinational Use of Anti-EGFR and Anti-Vascular Monoclonal Antibodies

The BOND-2 study is a phase II clinical trial of cetuximab, bevacizumab, and irinotecan compared with cetuximab and bevacizumab alone in irinotecan-refractory colorectal cancer. It showed that the time to tumor progression (TTP) is 7.3 months in patients receiving the combination treatment of cetuximab, bevacizumab, and irinotecan. The response rate is 37%, and the OS is 14.5 months. These response rates are superior to that of other studies involving patients with refractory mCRC (22). However, the subsequent phase III trial does not show the same results. PACCE is a phase III randomized open-label clinical trial to evaluate the efficacy of oxaliplatin or irinotecan plus bevacizumab with or without panitumumab as the first-line therapy in mCRC (23).

The results in the combination group do not indicate benefit in PFS and OS (PFS: 10.0 months vs.11.4 months; HR 1.27; OS: 19.4 *vs.* 24.5 months; HR, 1.43). CAIRO2 is a phase III randomized open-label clinical trial, which evaluates the efficacy and safety of bevacizumab and capecitabine/oxaliplatin with or without cetuximab as the first-line therapy in 755 mCRC patients (24). The efficacy endpoint showed a shortened PFS in the patients receiving the combination treatment of bevacizumab and cetuximab (9.4 months vs. 10.7 months; HR, 1.22). The overall incidence of grade 3/4 toxicity in the combination treatment group is significantly increased. Therefore, the current anti-EGFR drug in combination with macromolecule bevacizumab for the treatment of advanced CRC has not been approved (**Table 1**).

**Table 1:** Combination treatment of anti-EGFR and anti-VEGF drugs in patients with metastatic colorectal Cancer.

Trails	Phase	Arm	Number	(RR%)	P Value	Time (m)	P Value	OS (m)	P Value
Saltz LB (22)	II	CBI	43	37	NA	7.3 (TTP)	NA	14.5	NA
		CB	40	22		4.9		11.4	
Hecht JR (23)	IIIB	PBC	823	46	NS	10 (PFS)	NA	18.4	0.16
		BC	230	48		11.4		not reached	
Tol J (24)	III	CB*	378	50	0.49	10.7(PFS)	P=0.01	20.3	NS
		CBC*	377	52.7		9.4		19.4	

RR, response rate; TTP, time to tumor progression; OS, overall survival; CBI, cetuximab bevacizumab and irinotecan; CB, cetuximab and bevacizumab alone; PBC, bevacizumab and chemotherapy with panitumumab; BC, bevacizumab and chemotherapy; CB*, capecitabine oxaliplatin, and bevacizumab; CBC*, capecitabine oxaliplatin, and bevacizumab plus cetuximab; NA, not applicable; NS, not statistically significant.

## Clinical Trials on Combination Treatment of Anti-EGFR and Small-Molecule Anti-VEGF Drugs

Fruquintinib and regorafenib are all small-molecule anti-VEGF agents. Fruquintinib and regorafenib are VEGFR1/2/3 inhibitors with high selectivity (25), that inhibit tumor cell proliferation and angiogenesis (26). Clinical studies have indicated that bevacizumab-resistant CRC patients can be benefited from fruquintinib and regorafenib treatment (3–5), most likely associated with the fact that bevacizumab only targets VEGF1, and the bypass is activated. The resistance to bevacizumab is mostly attributed to the activation of other VEGFs. Therefore, switching to fruquintinib and regorafenib has some efficacy and been clinically validated. The macromolecular anti-vascular drug bevacizumab in combination with anti-EGFR drugs has no clinical effect in the treatment of CRC, which may be related to the single inhibitory target of bevacizumab and macromolecular monoclonal antibody. According to preclinical studies (12–14), anti-VEGF and anti-EGFR may have a synergistic effect. An exploratory study of regorafenib in combination with cetuximab in the treatment of advanced tumors has been performed. Early clinical studies and subsequent phase IB studies have shown a certain effective rate in the treatment of advanced tumors (27, 28). In the early clinical trial, one CRC patient achieves PR, and the subsequent phase IB study has reported that one CRC patient among five patients achieves PR. These two clinical studies have demonstrated a certain efficacy of regorafenib in combination with cetuximab in cancers. The patients can generally tolerate the toxic and side effects after dose adjustment. However, the patients enrolled in these two studies have diverse cancers. Therefore, the trials do not clearly reflect the efficacy of CRC patients.

Currently, there is no report on fruquintinib in combination with cetuximab in the treatment of mCRC. In this case, the patient with mCRC underwent the fourth-line treatment with cetuximab and regorafenib. The efficacy was SD and the PFS exceeded 8 months. No serious side effects were observed in this patient, and the tolerability was acceptable. Compared with the single-agent fruquintinib with a PFS of 3.71 months, the combinational treatment was more effective than treatment with fruquitinib alone, and it may help to reverse the resistance to chemotherapy, cetuximab and regorafenib. The reason might be partly attributed to the mutual synergy of anti-EGFR drug cetuximab in combination with the anti-vascular drug fruquintinib. The mechanism underlying the reversed resistance should be further explored. The major limitation of this study is that, this combinational use is in only one case study. The clinical effects of small-molecule anti-vascular drugs in combination with cetuximab in the treatment of CRC should be further investigated.

## Conclusion

Patients with advanced RAS/BRAF wild-type CRC after resistance to third-line therapy should consider using the combination of small-molecule antiangiogenic agents and anti-EGFR drugs.

## Author Contributions

HZ conceived and designed the study. YL drafted the manuscript. All authors contributed to the article and approved the submitted version.

## Funding

This work was supported by the Foundation of Guangdong Provincial Hospital of Chinese Medicine (2017KT1820).

## Conflict of Interest

The authors declare that the research was conducted in the absence of any commercial or financial relationships that could be construed as a potential conflict of interest.
